# Evaluation of a school-based depression prevention program among adolescents with elevated depressive symptoms: study protocol of a randomized controlled trial

**DOI:** 10.1186/s12888-016-1119-8

**Published:** 2016-11-16

**Authors:** Karlijn W. J. de Jonge-Heesen, Kim M. van Ettekoven, Sanne P. A. Rasing, Farina H. J. Oprins-van Liempd, Ad A. Vermulst, Rutger C. M. E. Engels, Daan H. M. Creemers

**Affiliations:** 1 GGZ Oost Brabant, P.O. Box 3, 5427 ZG Boekel, The Netherlands; 2 Utrecht University, P.O. Box 80125, 3508 TC Utrecht, The Netherlands; 3 Behavioural Science Institute, Radboud University Nijmegen, P.O. Box 9104, 6500 HE Nijmegen, The Netherlands; 4 GGD Hart voor Brabant, P.O. Box 3024, 5003 DA Tilburg, The Netherlands; 5 Trimbos Institute, P.O. Box 725, 3500 AS Utrecht, The Netherlands

**Keywords:** Prevention, Depression, Adolescents, School-based, Indicated, Resilience

## Abstract

**Background:**

Adolescents are at risk of developing depressive symptoms. Given the prevalence, recurrence and negative consequences of adolescent depression, it is crucial to implement prevention programs for high-risk adolescents. Prevention programs at an indicated level have shown to be successful in reducing depressive symptoms in adolescents. This study will evaluate the (cost)effectiveness of the prevention program ‘Op Volle Kracht (OVK 2.0)’ for adolescents with elevated depressive symptoms.

**Methods:**

We will perform a Randomized Controlled Trial (RCT) with an intervention and control condition to test the effectiveness of an indicated prevention program aimed at depression in adolescents. Adolescents in their second year of secondary education (11–15 year) will be screened for depressive symptoms. Those with heightened levels of depressive symptoms (CDI-2 ≥ 14) will be randomly assigned to the intervention (*N* = 80) or control group (*N* = 80). The participants in the intervention condition will receive a prevention program comprising eight meetings of 60 min each. The participants in the control condition will receive psycho-educational information. All participants and their parents will complete assessment at baseline, post-intervention, and 6-, 12- and 24- month follow-up. Primary outcome will be depressive symptoms. Additionally, the present study will identify mechanisms that mediate and moderate the program effects and test the effect of OVK 2.0 on secondary outcomes.

**Discussion:**

This paper describes a study designed to screen adolescents for depressive symptoms and offer them a prevention program to prevent the onset of depressive symptomatology. Adolescents in the intervention condition are expected to show lower levels of depressive symptoms at 12 month follow-up compared to adolescents in the control condition. If OVK 2.0 proves to be effective, the screening and intervention program could be implemented in schools on a large scale.

**Trial registration:**

Dutch Trial Register NTR5725. Date registered: 11^th^ of March 2016.

## Background

Depression is a major public health concern, causing an emotional burden for people and a considerable socio-economic burden for society. International studies show prevalence rates of depression between 2 and 5.6% in adolescents [1, 2]. A Dutch study showed that 5.6% of the adolescents experienced a depression before the age of 15 years [3]. Depressive symptomatology interferes with normal development and is associated with several negative outcomes, including other psychiatric disorders later in life [4], educational impairments [5], self-injury [6], and even suicide [7]. In addition to the individual consequences, depression has considerable economic and societal consequences. In 2003, healthcare costs for the treatment of depression were 660 million euros in the Netherlands [8]. In 2005, the Dutch healthcare costs for the treatment of depression increased to 773 million euros [9]. As the number of adolescents suffering from depression rises dramatically [10], this might an appropriate phase in life to start with prevention.

Depression is one of the most prevalent mental disorders among adolescents, but it is often difficult to observe and therefore not recognized [11]. Research shows that depressive symptoms are unidentified more often in adolescents than in adults [12]. Adolescence is characterized by fluctuating symptoms, mood reactivity, and prominence of irritability. Additionally, other impairments, such as eating problems, anxiety, unexplained physical symptoms, truancy, or a decline in academic performance are often present and they can cover up depressive symptoms [13]. This is concerning, as an early onset of depressive symptoms and the duration of unidentified and untreated depression are both risk factors for severe depressive disorders in later life [14]. Further, this shows that adolescence in particular is the time to prevent the development of serious depression disorders. However, as adolescents are less willing to seek help for their mental health problems, especially when they experience subclinical symptoms [15], there is a need for an early identification and prevention strategy.

Over the past years, several programs aimed to prevent depression among adolescents have been developed. These programs are based predominantly on the principles of cognitive behavioral therapy (CBT). In accordance with Beck’s cognitive theory, they assume that negative cognitions and cognitive distortions increase depressive symptoms and that reappraising and correcting misinterpretation diminish depressive symptoms [16]. In these prevention programs, adolescents learn to recognize cognitive biases and change negative thoughts into more helpful thoughts that make them feel better. The existing research shows that these programs have small to moderate effect sizes in reducing depressive symptoms [17, 18] or prevent the onset of a depression [19]. Prevention can be divided into three different levels; (1) universal prevention focused on the entire population, (2) selective prevention focused on high risk individuals, and (3) indicated prevention focused on individuals with early symptoms of a disorder [20]. Research to date shows that indicated prevention targeting adolescents with subclinical depressive symptoms proves to be more effective in preventing the development of adolescent’s depression compared to universal prevention programs [17, 18, 21].

In the Netherlands, the effectiveness of depression prevention is examined with the prevention program ‘Op Volle Kracht’ (OVK), which translates to ‘On Full Power’. OVK is the Dutch version of the Penn Resiliency Program [22] that has proven to be successful in reducing depressive symptoms [23]. This program was designed for adolescents aged 12 to 14, and it includes CBT, cognitive coping skills, and social skills. The effectiveness of OVK has been studied through randomized controlled trials at universal- selective-, and indicated- prevention level. Consistent with the previous analyses, OVK was not effective at universal level [24] and selective level [25]. However, OVK was effective at an indicated level among adolescents girls [26] when it included only the first eight lessons based solely on CBT techniques. This is in line with Stice et al. [21] who showed, that longer prevention programs lead to less positive outcomes. Moreover, the focus on CBT might be more effective than the combination of several techniques.

The present study proposes to screen adolescents for depressive symptoms and suicidal ideations, offer them a prevention program to prevent the onset of depression, and evaluate the (cost) effectiveness of this prevention program. To achieve these aims, this study will use the modified OVK version comprising 8 lessons of Wijnhoven et al. [26] that is adapted to an up to date version with a focus on high-risk adolescents and is therefore called OVK 2.0. The effectiveness of OVK 2.0 compared to psycho-education will be examined in a randomized controlled trial with follow-up assessments up to 24 months. An intensive collaboration between schools and (mental) health care organizations has already begun in a rural area in the south of the Netherlands to test the effectiveness of OVK 2.0. In addition to the primary aims of present study, factors that might influence the effectiveness of OVK 2.0 will be investigated. Although various studies examined the effectiveness of depression prevention programs, mechanisms underlying the effectiveness are still unknown. It is important to identify these factors, as this provides insight into the development of depressive symptoms among adolescents and directions for further improvement of the working mechanisms of prevention programs.

First, mediation effects will be investigated. Previous research showed that a ruminative response style worsens the depressive feelings [27]. Furthermore, it is crucial to investigate the effect of prevention on rumination and other cognitive coping styles, as this might influence the prevention effectiveness and the course of depressive symptoms. Another possibly mediating factor that will be included in this study is perfectionism. Adolescents with depressive symptoms are more likely to perceive that others have high standards for them; thus, they feel they must satisfy those standards [28]. CBT techniques might teach adolescents to cope with irrational perceptions, which might decrease their scores on perfectionism and depressive symptoms. In addition, the presence of negative life-events will be included as a mediator. Life events can be divided into dependent and independent life-events. Dependent life-events are mostly interpersonal (e.g,. conflicts; [29]). Independent life-events refer to events on which one has no influence (e.g*.,* divorce of parents). Individuals suffering from a depression experience more dependent negative events, and these events are highly predictive of depressive symptoms in adolescence [30]. A prevention program might increase coping skills, which might affect the presence of dependent life events that might in turn decrease depressive symptoms.

Second, certain moderators, such as age and gender, might influence the outcomes of this study; therefore, they will be examined. Based on previous meta-analyses of CBT prevention programs, larger effects are expected for girls and older adolescents [17, 21]. Adolescent girls are more likely to develop a major depression [31] and report greater depressive symptoms compared to adolescent boys (e.g., [32]). This might be the reason that girls are more susceptible for intervention effects. Additionally, older adolescents may struggle less to acquire the skills due to cognitive maturation [33]. It is important to understand the role of moderators to identify adolescents who benefit the most from the intervention and adolescents who are unlikely to benefit from the intervention [34].

Third, a prevention program might also affect secondary outcomes that are highly correlated with depressive symptoms, such as suicide risk. Since 2010, suicide has been the most significant cause of death among 15 to 29 years old individuals in the Netherlands [35]. Moreover, untreated depression is one of the most frequently reported risk factors associated with adolescents’ suicide (e.g., [7]). Strikingly, research shows that only a minority of the adolescents who committed suicide was receiving psychiatric treatment at the time of death [36]. The effect of OVK 2.0 on suicide risk might be relevant, as suicide has a massive influence on people and society. Other secondary outcomes included in the present study are anxiety [37], somatic complaints [38], and academic performance [5]. Additionally, cost-effectiveness, parents’ reports of depressive symptoms, and the prevention of a clinical depression are included.

## Methods

The study design will be reported in accordance with the CONSORT 2010 statement for reporting parallel group randomized trials [39]. The medical ethics committee CMO Region Arnhem-Nijmegen in The Netherlands approved this study (NL55328.091.15). The study is registered in the Dutch Trial Register for RCT’s (NTR5725).

### Design

The present study will be a non-blinded randomized controlled trial (RCT) with two conditions (intervention versus control). The participants in the intervention condition will receive a CBT-based prevention program and participants in the control condition will receive psycho-education. Participating schools are located in a rural region in the south of the Netherlands. Students in their second year of secondary school, from vocational training up to pre-university level, will be screened for depressive symptoms using the Dutch version [40] of the Childhood Depression Inventory 2 (CDI-2; [41]). This screening is part of a large health survey that is occupied by the public health service in schools (in Dutch: GGD). Adolescents with elevated symptoms of depression will be selected and recruited. Those who will be identified with suicidal ideation during the screening or at any time point during the study will be seen within 48 h by the public health service of school. Eventually, clinical referrals will be provided and these adolescents will be excluded from the intervention. However, they will be asked to complete the same set of questionnaires as the participants in the intervention and control condition to examine the effect of the screening and referral.

**Fig. 1:**
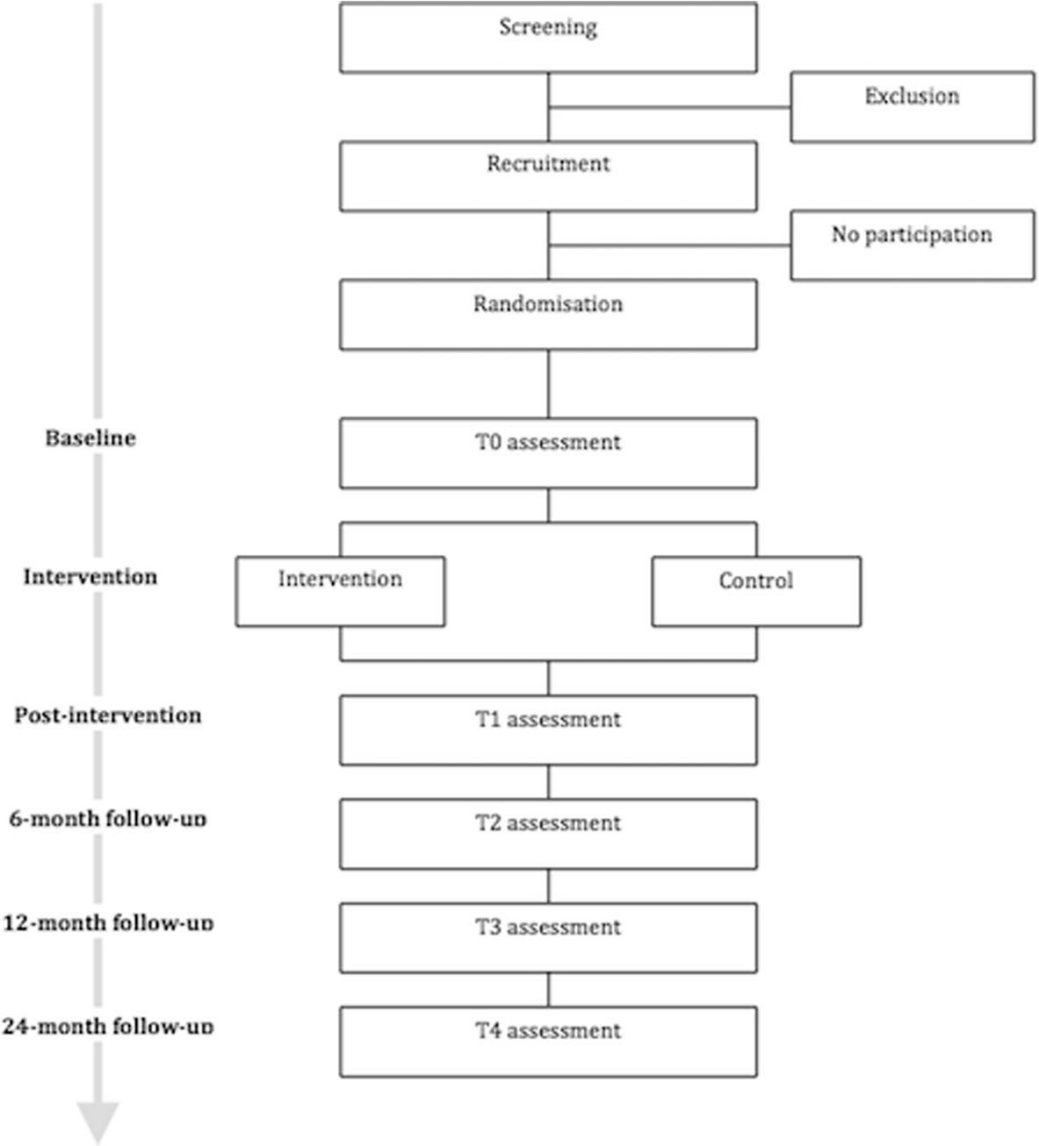
Schematic overview of the study design

After the screening and recruitment, participants will be randomly assigned to the intervention or control group. Adolescents and their parents will complete a set of questionnaires at baseline (T0). Furthermore, adolescents will undergo a semi-structured interview (ADIS-C) [42] to determine the presence of clinical depression. The assessments to evaluate the effects of the intervention will be conducted immediately after the intervention (T1) and at 6- (T2), 12-(T3), and 24- month follow-up (T4). The clinical interview will be repeated at 6-month follow-up. Figure 1 shows a schematic overview of the design of the present study.

If severe depressive symptoms will be identified during the 6-month follow-up interview, appropriate clinical referrals will be provided for adolescents in either condition. The severity of depressive symptoms will be determined on the clinical guidelines for depression and youth in the Netherlands [43]. The criteria include impairment in social functioning, the number of symptoms of a clinical depression according to the DSM-IV, suicidal ideation, psychotic symptoms, and course and characteristics of the depression.

### Participants’ eligibility

Adolescents with elevated depressive symptoms (score ≥ 14 CDI-2; [40]) are eligible for this study. Inclusion criteria are aged 11 to 15 years old and sufficient knowledge of the Dutch language. Exclusion criteria are the absence of parental permission and already undergoing a treatment for mood problems.

### Recruitment

Students who will meet the eligibility criteria (score ≥ 14 CDI-2) will receive verbal and written information about the study and written informed consent from adolescents and parents will be obtained. Subsequently, an independent researcher will randomly assign participants to one of the two conditions. Randomization will be carried out within schools to control for school characteristics.

Adolescents with suicidal ideation during the screening or at any time point within the study (score two on CDI-2 item: a desire to kill oneself, if given the chance and/or score ≥ 23 on the VOZZ-Screen) will be seen by a public health service professional at the school. Parents will be contacted and information about possible mental health care services will be provided.

### Sample size

The sample size is based on the expected difference (Cohen’s *d* = 0.25) in the primary outcome between the experimental and control condition at 12 months follow-up (based on a meta-analytic review; [17]). To detect significant differences between subjects (condition effect), a total sample size of 78 (39 in each condition) is needed, assuming type I error of 0.5 and type II error of .20 (power = .80). Potential loss of power due to data clustering has to be considered, since the intervention will be conducted in small groups, with the mean of seven children in each group. Therefore, the ICC (estimated at 0.07 based on the results of Wijnhoven et al. [26]) needs to be incorporated into the sample size calculation. The Variance Inflation Factor (VIF; [44]) equals 1.49, indicating that the sample size must be increased to 117. We intend to increase the sample size by 40% to compensate for the potential loss of power and drop-out, resulting in 160 participants (80 in experimental condition and 80 in control condition).

### CBT intervention

The intervention will be based on the principles of CBT. In the first lesson, the participants will learn about emotions and depressive feelings. The adolescents will learn to identify emotions and thoughts they experience. During this program, they will use a schedule to learn that activating events, beliefs, emotional consequences and behavioral consequences are related. In the second lesson, the adolescent will learn about the relationships among activating events, beliefs, and emotional consequences. Beliefs can be optimistic or pessimistic and play a major role in the emotional consequences. The adolescents will also learn to recognize pessimistic beliefs. In the third lesson, adolescents will learn how to recognize cognitive errors. In the fourth lesson, adolescents will learn to investigate their thoughts and to find evidence for and against their thoughts. In the fifth lesson, adolescents will continue to find evidence for and against their thoughts and will start to test whether their thoughts are actually true. In the sixth lesson, participants will investigate theirs thoughts by asking the question ‘what’s next?’. They will learn to question their thoughts by imagining the worst case scenarios of their thoughts. In addition, they will learn to create an action plan to prevent the worst case scenario from actually happening. In the seventh lesson, adolescents will learn to replace thoughts and prove that the alternative belief is true. The eight and last lesson is meant to finish the intervention in a fun way. Adolescents can share their experiences with the intervention and take a quiz on everything they learned. In addition to the lessons, adolescents will monitor and record their mood daily, during the intervention. Homework will include energizing assignments based on behavioral activation which are assumed to have a positive effect on adolescents’ mood (e.g., [45]).

OVK 2.0 is a modified and more up to date version of the OVK program. It consists of eight lessons of 60 min each instead of the original 16 lessons. The program will be based solely on CBT techniques, which will be covered during the first 8 lessons of OVK. Lessons 9 to 16 of the OVK program will focus on social and cognitive coping skills, self-esteem, problem solving, and decision making, which are not included in OVK 2.0. In the original OVK program, homework was used to practice techniques learned in the lessons. Homework in OVK 2.0 will include mood monitoring and energizing assignments. Additionally, multi-media sources, such as video fragments, and a quiz to which the adolescents will have to respond using their phones, will be used in OVK 2.0.

The program will be delivered by licensed psychologists that are also staff members at school, together with a co-trainer. This co-trainer could be someone of the collaborated (mental) health care organizations or a staff member at the school. They will receive a 3-day training program covering training in CBT skills, theoretical principles, and the intervention protocol. The training will be delivered by licensed and experienced psychologists. Trainers and co-trainers will receive a detailed manual of OVK 2.0 and are offered support by the research team. After each lesson, trainers will complete an integrity checklist to improve intervention fidelity.

### Psycho-education

The psycho-education condition consists of providing a brochure with information about depressive symptoms. Additionally, participants will receive two e-mails with useful tips to boost their mood and decrease depressive symptoms. For example, they are encouraged to do more physical exercises and to find a sport they might like.

### Study outcome measures

Table 1 shows an overview of the study outcome measures that will be assessed at each time point.

**Table 1:** Overview of the assessment

	Screening	T0	T1	T2	T3	T4
Adolescent						
Depression (CDI-2)	X	X	X	X	X	X
Depression disorder (ADIS-C)		X		X		
Suicide risk (VOZZ-Screen)		X	X	X	X	X
Health status ( EQ-5D-5 L)		X	X	X	X	X
Anxiety (STAI)		X	X	X	X	X
Somatic complains (CSI)		X	X	X	X	X
Perfectionism (MPS)		X	X	X	X	X
Coping (CERQ)						
Life-events (ALEQ-R)		X	X	X	X	X
*Parents*						
Depression (CDI-2)		X	X	X	X	X
Healthcare costs (TIC-P)		X	X	X	X	X
School						
Academic grades		X	X	X	X	X
Drop-out rates		X	X	X	X	X
Non-attendance		X	X	X	X	X
Truancy		X	X	X	X	X

### Screenings measures

To assess the eligibility, adolescents will be screened for depressive symptoms using the CDI-2 [40, 41]. The CDI-2 is a self-report questionnaire comprising 28 items, each consisting of three statements rated in severity from 0 to 2 (e.g., ‘I don’t feel alone’ = 0, ‘I often feel alone’ = 1, ‘I always feel alone’ = 2). Item 8 of the CDI-2 measures the presence of suicidal ideation on a three point scale (0 = I don’t think about ending my life, 1 = I think about ending my life, but I would never do it, 2 = I want to end my life). The CDI-2 will be used for screening purposes in accordance with the Dutch clinical guidelines for depression among youth [43].

### Primary outcome measure

***Depressive symptoms*** in children and adolescents will also be measured with the CDI-2 [40, 41], as described in previous section.

### Secondary outcome measures

***The presence of a clinical depression*** will be measured by the Anxiety Disorder Interview Schedule for Children (ADIS-C; [42]) during a clinical interview. This semi-structured diagnostic interview can be used to diagnose anxiety and comorbid disorders in 7 to 17 years old children. The interview will be administered by a qualified psychologist or by a master student under the supervision of a qualified psychologist. The present study will focus only on affective disorders. Participants will have to respond ‘yes’, ‘no’ or ‘different’ to standardized questions. The purpose of this interview will be to investigate whether children meet the criteria for depression. If participants meet the criteria for depression, the severity will be determined using the checklist of the clinical guidelines for depression and youth [43].

***Suicide risk*** will be measured using the Vozz-screen [46]. This questionnaire contains ten questions assessing thoughts and actions about life, self-harm, suicide, and suicidal ideations in the past 7 days. Items about participant’s life are rated on a 5-point scale from 1 (I totally agree) to 5 (I totally disagree) (e.g.*,* ‘I feel worthless’). Items about self-harm and suicide are rated on a 5-point scale from 1 (never) to 5 (very often) (e.g.*,* ‘I have harmed myself deliberately’). Items about suicidal ideation in the past 7 days are rated on a 5-point scale from 1 (never) to 5 (every day) (e.g.*,* ‘I thought that suicide would be a solution for my problems’). A score of 23 or above indicates high risk of suicide.

***Health care costs*** will measured with the child version of the Trimbos and Institute of Medical Technology Assessment Cost Questionnaire for Psychiatry (TIC-P; [47, 48]). This questionnaire contains 33 items designed to measure the direct and indirect costs of mental health problems. Parents register the number of hospital days, general practice visits, sessions with psychologists, and other relevant events in the past 3 months. The indirect costs include the number of ‘work loss’ days for parents and school absenteeism for adolescents.

***Health status*** will be measured using the EQ-5D-5 L questionnaire which provides a single index value for health status that can be used to investigate the cost-effectiveness of the intervention [49]. It comprises five dimensions: mobility, self-care, usual activities, pain/discomfort, and anxiety/depression. Every dimension contains five statements, and participants are asked to rate their health on each dimension by choosing the statement that fits them the best. The total score can be compared with the health status by the general population [50], and for each health status, a quality of live score can be calculated. This score can vary from -0.59 (worst possible health status) to 1 (best possible health status). These scores will be used to calculate the Quality Adjusted Life Years (QALY).

***Anxiety*** will be measured with the State-Trait Anxiety Inventory (STAI; [51]). This self-report questionnaire contains 20 items measuring state anxiety. Items are rated on a 4-point scale, with scores ranging from 0 (almost never) to 3 (almost always) (e.g.*,* ‘I feel nervous’).

***Academic performance,*** including academic grades, drop-outs, non-attendance, and truancy will be obtained in collaboration with the schools.

***Somatic complains*** will be measured with the 35-item Children’s Somatization Inventory (CSI; [45]). Adolescents have to rate whether they have been bothered by somatic symptoms in the last 2 weeks on a 5-point scale ranging from 0 (no suffering) to 4 (many suffering) (e.g*.,* ‘Headache’ or ‘Fainting spells’).

***Perfectionism*** will be measured using the Frost Multidimensional Perfectionism Scale (MPS; [52, 53]). This 35-item questionnaire consists of six dimensions of perfectionism: personal standard (e.g., ‘I set higher goals than most people’), concern over mistakes (e.g., ‘I hate being less than the best at things’), organization (e.g., ‘I am a neat person’), doubt about actions (e.g., ‘I usually have doubts about the simple everyday things I do’), parental expectations (e.g.*,* ‘My parents set very high standards for me’), and parental riticism (e.g.*,* ‘My parents never tried to understand my mistakes’). Adolescents will have to rate statements on a 5-point scale ranging from 1 (strongly disagree) to 5 (strongly agree).

***Cognitive coping strategies*** will be measured with the Cognitive Emotion Regulation Questionnaire (CERQ; [54]). This questionnaire consists of nine subscales comprising of 4 items each. Adolescents will have to rate on a 5-point scale ranging from 1 (not at all) to 5 (a lot) to what extent they had used this strategy in response to stressful events. The CERQ contains the following subscales: catastrophizing (e.g., ‘Again and again, I think about how terrible it all is’), acceptance (e.g., ‘I think that I can’t do anything about it’), other blame (e.g., ‘I think that others are to blame’), positive refocus (e.g.*,* ‘I think about nicer things that have nothing to do with it’), positive reappraisal (e.g.*,* ‘I think that I can learn from it’), refocus on planning (e.g.*,* ‘I think of how I can best cope with it’), putting into perspective (e.g*.,* ‘I think that worse things can happen’), rumination (e.g., ‘Again and again, I think about how I feel about it’), and self-blame (e.g.*,* ‘I think that it’s my own fault’).

***Negative life events*** will be measured with the Dutch translation of the Adolescent Live Event Questionnaire-Revised (ALEQ-R; [25, 30]). The questionnaire contains 29 items assessing how often the dependent and independent negative life events occurred during the past 3 months on a five-point scale ranging from 0 (never) to 5 (always) (e.g., ‘You got in trouble with the teacher or principal’).

***Depressive symptoms according to parents*** will be measured with the Dutch translation of the CDI-2 [40, 41]. The questionnaire contains 17 items measured on a 4-point scale from 0 (not at all) to 3 (almost always) (e.g., ‘My child seems lonely’). Parents will have to rate the extent to which the items are in accordance with their child’s thoughts and feelings.

### Data analysis/statistical analysis

The data will be analyzed according to the intent-to-treat principle. Multiple imputations will be used to handle missing values at post-intervention and follow-up measurements. The results of the study will be reported in accordance with the CONSORT Statement [39].

To test the differences in the development of depressive symptoms between participants in the experimental and participants in the control group, a 5 (within subjects: pre, post, 6-, 12-, 24 follow-up) by two (experimental vs. control) two-way mixed ANOVA (repeated measures) will be conducted with depressive symptoms (adolescent report) as the dependent variable. To examine and test change in depressive symptoms over time, we will use Latent Growth Curve Modeling with Mplus [55]. Growth parameters (intercepts, linear slopes and possible quadratic terms) will be estimated and condition will be included as a predictor to test the effect of condition on these parameters. The Full Information Maximum Likelihood estimator is sufficient to deal with missing values [56, 57].

To test the mediating role of perfectionism, life events and cognitive coping style, mediation analyses will be performed in Mplus [55]. The indirect effects will be tested with bootstrap methods. To examine how parameters moderate the effect of condition on the growth parameters of depressive symptoms, the moderators will be included as covariates separately. The treatment effects of OVK 2.0 on secondary outcomes will be investigated in the same way as the primary study parameter, that is, depressive symptoms. Additionally, remission rates of the depression disorders that were diagnosed at baseline (with ADIS-C) will be calculated at 6-months follow-up, and Chi-square (*χ* ^2^) tests will be conducted to compare remission rates between the experimental and control group.

In the economic evaluation study, we will use incremental-cost ratios in which we compare, incremental costs and incremental outcomes of the OVK 2.0 intervention in relation to psycho-education (control group). Arithmetic mean cost differences are the most appropriate measures to describe cost data. Because cost data do not conform to the assumptions of standard statistical test we will use bootstrapping resample methods [58] to test statistical differences between the intervention and the control group. The maximum amount of budget that society is prepared to pay to improve the treatment effectiveness determines the choice of treatment.

## Discussion

The present study protocol gives an overview of a RCT on the effect of OVK 2.0 on depressive symptoms in adolescents in a school-based setting. The primary aim is to investigate the effectiveness of an indicated depressive prevention program OVK 2.0 in adolescents. It is hypothesized that adolescents in the intervention condition will show less depressive symptoms during follow-up assessments, compared to the adolescents in the control condition receiving psycho-educational information. The secondary and third aims are to investigate factors that possibly mediate (cognitive coping style, perfectionism, negative life-events) and moderate the effect (age and gender) of the prevention program. The fourth and last aim is to test the effect of OVK 2.0 on secondary outcomes. The present study will investigate whether OVK 2.0 affects the following outcomes: cost-effectiveness, suicide risk, anxiety, somatic complaints, academic performance, adolescent’s depressive symptoms according to parents, and the presence of a clinical depression.

### Strengths and limitations

One of the strengths of this study is that it will include long term follow-up assessments of up to 24 months, providing the opportunity to evaluate the long-term effects. Second, the program will be implemented in all secondary schools in a rural region in The Netherlands with a strong collaboration between schools’ and (mental) health organizations. A meta-analysis of Brunwasser and Garber [59] on the effectiveness of programs for the prevention of youth depression revealed the need for studies conducted in real-life conditions. The present study will be relevant to the discussion about practical implementation. Third, parents’ assessments of adolescents’ depressive symptoms and a clinical interview will be conducted to supplement the information gathered exclusively from self-reports. The addition of a semi structured interview gives the opportunity to identify the presence or absence of a clinical depression in a more objective manner and enable us to investigate whether the intervention is successful in the prevention of a depression disorder. Fourth, in contrast to most RCT studies, not only the effectiveness of the prevention program will be evaluated, but also cost-effectiveness will be taken into account. This will be of great importance for the rising healthcare costs.

Several limitations of this study must be noted. The project will focus on adolescents with elevated depressive symptoms only. This might lead to a stigmatization effect during the process of identification and participation in the prevention program. Moreover, the study will be conducted in a specific region in The Netherlands, which may limit the generalizability of the results to other regions in The Netherlands.

### Implications for practice

If the OVK 2.0 program proves to be effective in preventing depressive symptoms in adolescents, it will have positive effects on the adolescents and society in general. Adolescents will experience less depressive symptoms, and the implementation of early identification and prevention could reduce the number of adolescents with a clinical depression. In addition, if the prevention program proves to be cost-effective, it will give the opportunity to lower health care costs associated with depression. Furthermore, the results of the study can increase our knowledge of the mechanisms that underlie the development of depressive symptoms in adolescents. This would enable us to improve prevention programs for adolescents with depressive symptoms. Additionally, OVK 2.0 might affect other depression-related symptoms, such as anxiety or somatic complaints, which will further benefit adolescents’ (mental) health. Lastly, OVK 2.0 might be a suitable example for early detection and treatment that could be easily implemented in the school system, which might be an answer to the increasing number of young people experiencing depression.

## Abbreviations

ADIS-C: Anxiety disorders interview schedule for children
ALEQ-R: Adolescent live event questionnaire-revised
CBT: Cognitive behavioral therapy
CDI-2: Children’s depression inventory 2
CERQ: Cognitive emotion regulation questionnaire
CSI: Children’s somatization inventory
MPS: Frost multidimensional perfectionism scale
OVK: Op Volle Kracht
RCT: Randomized controlled trial
STAI: State trait anxiety inventory
TIC-P: Trimbos and Institute of Medical Technology Assessment Cost Questionnaire for Psychiatry
